# Multidrug-Resistant *Salmonella enterica*, Democratic Republic of the Congo

**DOI:** 10.3201/eid1810.120525

**Published:** 2012-10

**Authors:** Marie-France Phoba, Octavie Lunguya, Daniel Vita Mayimon, Pantaléon Lewo di Mputu, Sophie Bertrand, Raymond Vanhoof, Jan Verhaegen, Chris Van Geet, Jean-Jacques Muyembe, Jan Jacobs

**Affiliations:** National Institute for Biomedical Research, Kinshasa, Democratic Republic of the Congo (M.-F. Phoba, O. Lunguya, J.-J. Muyembe);; University Hospital of Kinshasa, Kinshasa (M.-F. Phoba, O. Lunguya, J.-J. Muyembe);; Saint-Luc Hospital, Kisantu, Democratic Republic of the Congo (D.V. Mayimona, P. Lewo di Mputu);; Institute of Public Health, Brussels, Belgium (S. Bertrand, R. Vanhoof);; University Hospital Leuven, Leuven, Belgium (J. Verhaegen, C. Van Geet);; and Institute of Tropical Medicine, Antwerp, Belgium (J. Jacobs)

**Keywords:** Salmonella, bacteria, outbreak, Democratic Republic of the Congo, multidrug resistance, pediatric, epidemic, bacteremia, antimicrobial resistance, *Suggested citation for this article*: Phoba M-F, Lunguya O, Mayimona DV, Lewo di Mputu P, Bertrand S, Vanhoof R, et al. Multidrug-resistant *Salmonella enterica*, Democratic Republic of the Congo [letter]. Emerg Infect Dis [Internet]. 2012 Oct [*date cited*]. http://dx.doi.org/10.3201/eid1810.120525

**To the Editor:** *Salmonella enterica* serotype Typhi and the nontyphoid *S. enterica* (NTS) are leading causes of bacteremia in sub-Saharan Africa, but little information is available from central Africa (*1*,*2*). We describe an epidemic increase of *S. enterica* bacteremia in Kisantu in southwestern Democratic Republic of the Congo (DRC).

The Hospital of Saint Luc in Kisantu is a 274-bed referral hospital serving a community of 150,000 inhabitants. As part of an ongoing microbiological surveillance study in DRC (*3*), we identified pathogens grown from blood cultures (BacT/ALERT; bioMérieux, Marcy L’Etoile, France) and assessed them for antimicrobial drug susceptibility (Vitek II system; bioMérieux) (*4*) and serotype (Sifin, Berlin, Germany). We determined MICs for nalidixic acid, ciprofloxacin, and chloramphenicol using the Etest macromethod (bioMérieux). For salmonella isolates, we defined decreased ciprofloxacin susceptibility as an isolate with an MIC >0.064 mg/L (*5*) and multidrug resistance (MDR) as co-resistance of the isolate to ampicillin, chloramphenicol, and trimethoprim/sulfamethoxazole (*6*). Screening for mutations causing decreased ciprofloxacin susceptibility included assessment of the quinolone resistance–determining regions of the *gyrA*, *gyrB*, and *parC* genes and the plasmid-mediated *qnrA*, *qnrB*, and *qnrS* genes (*7*). Multilocus variable-number tandem-repeat analysis was performed on a subset of 37 *S. enterica* ser. Enteritidis isolates (*8*).

The pathogens isolated were *S. enterica* ser. Typhi (n = 17, 14.4%), Enteritidis (n = 79, 67.0%), and Typhimurium (n = 22, 18.6%). The increased incidence of *S. enterica* bacteremia was caused by an increased incidence of *S. enterica* ser. Enteritidis infection from 1 and 2 isolates reported in 2008 and 2009, respectively. The rate of infection by serotypes Typhi and Typhimurium had remained constant during this period.

During September 2010–May 2011, the proportion of pathogens isolated from blood cultures increased to 53.2% (197/370), compared with 19.7% (63/319) and 25.2% (85/328) for 2008 and 2009, respectively (p<0.001). *S. enterica* isolates represented 59.9% (118/197) of pathogens, compared with 53.3% (70/131) and 30.9% (84/272) for the same months during 2008–2009 and 2009–2010, respectively (p<0.001). Of 118 *S. enterica* samples isolated, 89 (75.4%) were isolated from specimens from children <5 years old and 17 (15.3%) from children 5–10 years old. Clinical signs and symptoms were nonspecific; malaria and gastrointestinal infection were the leading diagnoses on admission. Data for in-hospital deaths (retrieved for 87 patients) revealed case-fatality rates of 23.0% (17/74) for children <5 years old, compared with 1 in 10 patients 5–10 years old. Because of the retrospective nature of the study, it was not possible to assess population incidence rates, and we had no estimates of the number of children who were referred to the hospital but died before reaching the emergency department. There was no apparent geographic clustering, but the epidemic coincided with the onset of the rainy season, which had started late and had unusually heavy rainfall.

All NTS isolates were MDR; 1 (1.3%) *S. enterica* ser. Enteritidis and 1 (4.5%) *S. enterica* ser. Typhimurium isolate had additional decreased ciprofloxacin susceptibility. Most (16/17, 94.1%) *S. enterica* ser. Typhi isolates were resistant to amoxicillin and trimethoprim/sulfamethoxazole, 4 (23.5%) and 7 (41.2%) were MDR and had decreased ciprofloxacin susceptibility, respectively. Three combinations of resistance genes encoding decreased ciprofloxacin susceptibility were found (Table). No resistance to cefotaxime was observed. Multilocus variable-number tandem-repeat analysis typing of the *S. enterica* ser. Enteritidis isolates revealed 2 major profiles (differing in 3 tandem repeats in 1 locus) and 3 minor profiles (differing from the major profiles by 1 tandem repeat at 1 and 2 loci, respectively). Considering the long sample period, we concluded that 1 clonal type had caused the infections in which *S. enterica* ser. Enteritidis was isolated.

**Table:** MICs and genetic analysis of *Salmonella enterica* isolates with decreased ciprofloxacin susceptibility, Democratic Rebublic of the Congo

Serotype	MIC, mg/L		No. isolates	Mutations
	Nalidixic acid	Ciprofloxacin		
Enteritidis	128	0.094	1	Asp82-Asn in gyrA + *qnrB*
Typhimurium	>256	0.125	1	Asp87-Tyr in gyrA + *qnrB*
Typhi	>256	0.19–0.25	7	Ser83-Phe in *gyrA*

A recent literature review of bacteremia reported aggregated data from 16 studies from eastern (Kenya, Tanzania), western (the Gambia), and southern Africa (Malawi, Mozambique) (*1*). *S. enterica* ser. Typhi and NTS represented 0–42% and 9%–84%, respectively, of associated pathogens. The study reported that most NTS isolated were of the serotypes Enteritidis and Typhimurium. A sequential occurrence of disease caused by NTS serotypes similar to that in this study was recorded in Malawi (*6*).

The reservoir of NTS in sub-Saharan Africa remains unclear; person-to-person and zoonotic transmissions have been postulated (*1*,*2*). The coincidence of the onset of the epidemic described in this study with the start of the rainy season is a known phenomenon and might be related to increased incidences of malaria and malnutrition (*9*) and to contamination of the surface waters caused by floods after heavy rainfalls (*2*).

Case-fatality rates in this study were similar to those reported previously from sub-Saharan Africa, describing mortality rates up to 27% (*2*), irrespective of the serotype involved. The MDR rates among NTS and among *S. enterica* ser. Typhi in this study are among the highest reported in sub-Saharan Africa (*10*). The finding of decreased ciprofloxacin susceptibility among isolates in a rural area in DRC highlights the need for surveillance of antimicrobial drug resistance of *S. enterica* isolates.
